# Plant Natural Compounds in the Treatment of Adrenocortical Tumors

**DOI:** 10.1155/2021/5516285

**Published:** 2021-09-17

**Authors:** Jacopo Manso, Javad Sharifi-Rad, Wissam Zam, Patrick Valere Tsouh Fokou, Miquel Martorell, Raffaele Pezzani

**Affiliations:** ^1^Endocrinology Unit, Department of Medicine (DIMED), University of Padova, Via Ospedale 105, Padova 35128, Italy; ^2^Phytochemistry Research Center, Shahid Beheshti University of Medical Sciences, Tehran, Iran; ^3^Facultad de Medicina, Universidad del Azuay, Cuenca, Ecuador; ^4^Analytical and Food Chemistry Department, Faculty of Pharmacy, Tartous University, Tartous, Syria; ^5^Faculty of Science, University of Bamenda, Bamenda-Bambili, Po. Box 39, Cameroon; ^6^Department of Nutrition and Dietetics, Faculty of Pharmacy, Centre for Healthy Living, University of Concepción, Concepción 4070386, Chile; ^7^Universidad de Concepción, Unidad de Desarrollo Tecnológico, UDT, Concepción 4070386, Chile; ^8^Phytotherapy Lab, Endocrinology Unit, Department of Medicine (DIMED), University of Padova, via Ospedale 105, 35128 Padova, Italy

## Abstract

Plant natural products are a plethora of diverse and complex molecules produced by the plant secondary metabolism. Among these, many can reserve beneficial or curative properties when employed to treat human diseases. Even in cancer, they can be successfully used and indeed numerous phytochemicals exert antineoplastic activity. The most common molecules derived from plants and used in the fight against cancer are polyphenols, i.e., quercetin, genistein, resveratrol, curcumin, etc. Despite valuable data especially in preclinical models on such compounds, few of them are currently used in the medical practice. Also, in adrenocortical tumors (ACT), phytochemicals are scarcely or not at all used. This work summarizes the available research on phytochemicals used against ACT and adrenocortical cancer, a very rare disease with poor prognosis and high metastatic potential, and wants to contribute to stimulate preclinical and clinical research to find new therapeutic strategies among the overabundance of biomolecules produced by the plant kingdom.

## 1. Introduction

The use of plants and plant-derived compounds dates back to prehistory. Prehistoric humans probably learned the beneficial use of plant natural substances from animals and over the years; they refined the curative technique applying new preparative methods (drying, boiling, concentration, collection period, etc.) in addition to the simple ingestion of fresh plants. Even at the present day, plants remain a wide and valuable source of natural compounds that can sometime become new therapeutic tools or even drugs, given the extensive range of diversity of phytochemical structures [1]. Plant natural products play a key role in different human diseases, including cancer. There are numerous research studies that reported the efficacy and safety of phytochemicals and about one-quarter of drugs derive from the plant kingdom [2, 3]. Moreover the first anticancer drugs used in clinics were alkaloids from *Taxus baccata* L. and *Vinca* spp. [4], two well-known plants for their therapeutic properties. Now such drugs are used to treat numerous tumors worldwide and represent an appreciated tool in the fight against cancer [5, 6]. Also endocrine neoplasms as adrenocortical tumors (ACT) and, in particular, the rare and lethal adrenocortical cancer (ACC) can be a potential target for plant natural compounds. The aim of this review is to recapitulate available research on phytochemicals used against ACT and ACC, searching for different databases, such as Embase, Google Scholar, Ovid, PubMed, SciFinder, Science Direct, Scopus, and Web of Science. The search strategy was based on the combination of different keywords, such as phytochemical (main categories, carotenoids, nitrogen-containing compounds, organosulfur, alkaloids, phenolic acids, flavonoids), plant natural compounds, adrenal, tumor, adrenocortical tumor, and adrenocortical cancer/carcinoma. Only articles in English language have been selected, excluding patents and symposium or congress papers.

## 2. Adrenocortical Tumors

**Figure 1:**
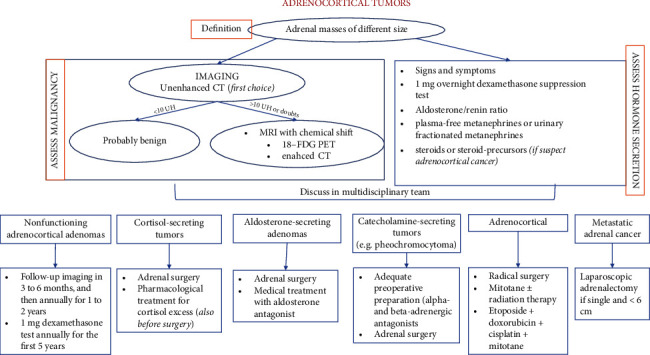
Diagnostic and therapeutic flow chart of adrenocortical tumors. 18-FDG PET, positron emission tomography with 18F-2-deoxy-d-glucose; CT, computed tomography; MRI, magnetic resonance imaging; HU, Hounsfield units.

Adrenocortical tumors (ACT) are adrenal masses of different sizes, mainly benign in nature and discovered incidentally (so-called “incidentaloma”) or due to signs and symptoms of hormonal secretion or compression. The prevalent etiology of adrenocortical tumors is nonfunctioning adrenocortical adenomas, which accounts for about 80% of cases [7]. Nonetheless, every patient should undergo a careful and comprehensive evaluation in order to identify hormone-secreting tumors (e.g., cortisol-secreting, aldosterone-secreting, or catecholamine-secreting tumors) or malignant tumors (e.g., adrenocortical carcinoma or metastatic adrenal cancer), which require surgery or medical treatment.  Figure 1 summarizes available data on ACT.

### 2.1. Imaging

The prevalence of adrenal nodules is between 4 and 10% on computed tomography (CT) series depending on age [8, 9]. In addition to the detection of adrenal lesions, imaging techniques are essential for distinguishing benign from malignant masses. Unenhanced CT is the most valuable imaging technique. In fact, it allows recognize benign lesions through the evaluation of tissue density measured in Hounsfield units (HU). Generally, an adrenocortical tumor with an HU of ≤10 is a lipid-rich tissue and thus considered benign [10]. Contrast-enhanced CT is useful for the evaluation of washout in indeterminate adrenal tumors. Malignant tumors enhance rapidly but have a slower washout of contrast medium; consequently an absolute washout of >60% in the medium phase strongly suggests a benign adenoma [11, 12].

Magnetic resonance imaging (MRI) has the advantage to avoid radiation exposure. The MRI technique of chemical shift is most commonly used to discriminate the benign masses from the malignant ones: the loss of signal on opposed phase imaging is indicative of an adrenal benign adenoma [13, 14].

Positron emission tomography with 18F-2-deoxy-d-glucose (18-FDG PET) predominantly combined with CT is mainly used for the detection of malignant tumors, which shows increased glucose metabolism [15, 16].

The lesion size is a well-known predictor of malignancy. The 4 cm cutoff is generally adopted also in the latest guidelines, with a sensitivity of about 93% for malignancy, but with a poor specificity of 42% [7, 17]. Indeed, the guidelines recommend surgery for adrenal tumors >4 cm, mainly due to the extremely poor prognosis and highly aggressive nature of ACC [18].

### 2.2. Cortisol-Secreting Tumors

Cortisol-secreting tumors are responsible for the clinical scenario of Cushing's syndrome. About 5–20% of all incidentalomas have some degree of autonomous cortisol secretion [19]. The symptoms and signs of exaggerated cortisol action are muscle wasting, relatively thin limbs, ecchymoses, thin skin, poor wound healing, striae (purple or “violaceous” rather than white), thin (osteoporotic) bones that easily fracture, diabetes mellitus, central obesity, rounded (“moon”) face, “buffalo hump,” susceptibility to infection, predisposition to gastric ulcer, hypertension, disturbance of menstrual cycle, symptoms overlapping with polycystic ovarian syndrome, and mood disturbance (depression, psychosis) [20]. There are three tests to screen for hypercortisolism: 24-hour urinary free cortisol test, late-night salivary cortisol test, and 1 mg overnight dexamethasone suppression test. The last one is the recommended test for all adrenocortical tumors [18]. Serum cortisol levels post dexamethasone ≤50 nmol/L without cortisol-related comorbidities exclude autonomous cortisol secretion [18, 21]. Serum cortisol levels post dexamethasone between 51 and 138 nmol/L should be considered “possible autonomous cortisol secretion” (previously known as subclinical hypercortisolism) and >138 nmol/L indicate “autonomous cortisol secretion” (or overt hypercortisolism) [18, 22]. However, there are a lot of conditions or drugs that can affect the results of the suppression test, e.g., estrogen therapies or malabsorption disease [23]. 24-hour urinary free cortisol and late-night salivary cortisol tests are useful in the doubtful cases or as confirmatory tests of hypercortisolism. The presence of an overt hypercortisolism is always an indication for adrenalectomy. Laparoscopic surgery versus open adrenalectomy is still debated; nevertheless, recent guidelines suggest (a) laparoscopic surgery in patients with unilateral adrenal tumor with radiological findings suspicious of malignancy and a diameter of ≤6 cm but without evidence of local invasion and (b) open procedure if there are also signs of local invasion [18]. If surgery is not feasible, pharmacological treatment for cortisol excess is available and can also be used before surgery to ameliorate the outcome [24].

### 2.3. Aldosterone-Secreting Tumors

Aldosterone-secreting adenomas represent between 1 and 3% of all adrenal incidentalomas. Aldosterone-secreting tumors cause the so-called “Conn's syndrome” or primary hyperaldosteronism, which is characterized by resistant hypertension and hypokalemia with an abnormal aldosterone/renin ratio [19]. Due to the low rate of aldosterone secretion, recent guidelines recommend the use of aldosterone/renin ratio to exclude primary aldosteronism only in patients with adrenal tumors and concomitant hypertension or unexplained hypokalemia [7]. When testing the aldosterone/renin ratio, potassium should be adequately corrected and drugs that can interfere with aldosterone and renin levels (e.g., aldosterone antagonists) should be avoided [25]. In case of abnormal aldosterone/renin ratios, patients should undergo at least one confirmatory test. When feasible, adrenalectomy is the gold standard; otherwise medical treatment with aldosterone antagonists should be initiated in order to control hypertension and hypokalemia. Even patients with a single adrenal mass at imaging technique could have bilateral aldosterone secretion [26]. Therefore, adrenal venous sampling (AVS) is the gold standard in distinguishing unilateral from bilateral secretion and most endocrine surgeons recommend AVS prior to adrenalectomy [27].

### 2.4. Catecholamine-Secreting Tumors

Facing with an adrenal tumor needs a plasma-free metanephrines or urinary fractionated metanephrines dosage, even if it appears to be cortical, to exclude a pheochromocytoma. This screening is safe and cost-effective [7, 28]. For detailed description, we refer to the recent guidelines of different societies [28].

### 2.5. Nonfunctioning Adrenocortical Adenomas

Nonfunctioning adrenocortical adenomas represent the vast majority of adrenocortical tumors and account for about 80% [7]. There are conspicuous differences between the most recent American and European guidelines in the management of this kind of adrenal tumors. The American ones published in 2009 recommend that silent adrenocortical tumors <40 mm with clear benign imaging features should undergo follow-up imaging in 3 to 6 months, and then annually for 1 to 2 years. Indeed, they reported a risk of an increase of 14% at 2 years and 29% at 5 years. Due to the risk (47%) of becoming hormonally active with a great prevalence of “possible autonomous cortisol secretion,” they also recommend hormonal evaluation annually for the first 5 years [29]. Differently, the European Society of Endocrinology Clinical Practice Guidelines do not recommend imaging follow-up in silent adrenal tumors <40 mm without malignant or suspicious for malignancy radiological features; furthermore, the guidelines also do not recommend repeating the hormonal assessment unless clinical signs of excess develop [7].

### 2.6. Metastatic Adrenal Cancer

In patients with a prior cancer history, the possible secondarism of an adrenocortical lesion should always be kept in mind [30]. The primary cancers that more frequently metastasize to adrenal glands are lung, breast, colon, and melanoma [31]. Bilateral adrenal metastasis rarely causes adrenal insufficiency [7]. A single adrenal metastasis may often be treated by laparoscopic adrenalectomy if it is <6 cm, and extended survival or even cure could be achieved [32–35].

## 3. Adrenocortical Cancer

ACC is a rare but extremely aggressive malignancy with poor prognosis [36, 37]. To date, radical (R0) surgery is the best treatment and the only one that can have a curative aim [38]. However, ACC shows a very high rate of recurrence (40–65% at 2 years) and the emergence of distant metastasis is frequent also in patients with R0 [39, 40]. Thus, ACC has a survival rate of 50% after 5 years and adjuvant therapy plays a pivotal role in the management of this malignancy [41, 42]. Hypersecretion of adrenal steroids is present in about 60% of cases with a predominance of hypercortisolism and hyperandrogenism in women. Estrogen-secreting ACC is rare, but if hyperestrogenism is present, it is pathognomonic for ACC [43, 44]. Of note that nonsecretory ACCs actually produce steroids or steroid precursors in at least 95% of cases when urinary metabolites are investigated with more sensitive methods [45]. Unenhanced CT or MRI is often enough to distinguish between benign and malignant adrenal masses, but in doubtful cases, enhanced CT or 18-FDG PET may be useful [12, 46–48]. The ENSAT tumor stage is a staging system proposed by the European Network for the Study of the Adrenal Tumors (ENSAT) that guarantees a better prognosis than the previous TNM classification [49].

Unfortunately, most of the recommendations in ACC therapy derive from retrospective studies or expert opinions due to the rarity of the disease. The best ACC treatment is essentially based on the feasibility of radical surgery [50]. About 50% of ACCs that have been completely surgically removed (R0) relapse till a patient's exitus [51–53]. In the most recent guidelines by the European Society of Endocrinology (ESE), mitotane remains the main drug for primary or adjuvant therapy for recurrent or relapsing disease, used alone or combined with other chemotherapeutics [54]. The ESE defines patients at high risk of recurrence after R0 surgery on the basis of ENSAT tumor stage III or IV or Ki67 > 10% and suggests adjuvant mitotane treatment in this group. Differently in low-intermediate risk of recurrence after R0 surgery, adjuvant mitotane remains an option to be discussed on individual basis [54]. In case of nonradical surgery (Rx o R1), the ESE suggests adjuvant mitotane treatment plus radiation therapy. The subsequent follow-up must be modulated according to the development of relapses. In advanced ACC, chemotherapy associated with mitotane is the standard of cure in combination with debulking surgery, but the outcomes remain little motivating, owing to the scarce response with no impact on overall survival (OS) and the significant toxicity [55]. The real utility of radiotherapy and other locoregional treatment is still debated [56]. The etoposide, doxorubicin, cisplatin plus mitotane (EDP-M) regimen represents the most widely accepted treatment for advanced ACC, being validated by a randomized controlled trial [55]. In case of disease progression under EDP-M regimen, additional therapies (other chemotherapy or minimally invasive procedures) or enrollment in experimental clinical trials should be consider [54].

## 4. Cell and Animal Models of Adrenocortical Carcinoma

The human adrenal cortex is a complex endocrine organ that secretes mineralocorticoids, glucocorticoids, and adrenal androgens that arise from morphologically and biochemically distinct zones of the adrenal gland [57]. Indeed, the most common form of endocrine hypertension results from the excessive secretion of aldosterone from the adrenal. However, understanding the underlining molecular mechanism of aldosterone synthesis and release is hampered by the lack of a suitable human adrenocortical cell line that reflects the molecular retaining ability to produce any of the major adrenal steroid products that faithfully reflect aldosterone-producing adenomas [57–59]. As well, clinical translation of novel therapeutic strategies for patients with ACC often fails [60] especially in children with advanced ACC where conventional chemotherapeutic agents have shown limited utility and efficacy [61]. These disappointing results indicate that the current tumor models only poorly reflect relevant pathophysiology and, thereby, do not predict clinical applicability of novel pharmacological approaches [60]. Overall, the development of novel anticancer agents heavily relied on xenograft models of human cancers. Nonetheless, they have specific limitations and variables, such as tumor origin, subculturing, genetic background, immune-competence of the murine host, hormonal activity, or site of implantation. These have to be individually taken into account for the design of preclinical studies on ACC. Cell-culture-derived xenograft models allow the establishment of metastatic or orthotopic models and the use of transfectants expressing luciferase or green fluorescent protein suitable for an *in vivo* imaging system [62]. Even if commonly available xenografts such as NCI-H295R, Y1, and SW-13 are productive, they derived from long-term *in vitro* culture susceptible to alteration of their original biological properties through selection pressure in cell cultures compared to the original patient tumors [63]. To overcome this limitation, patient-derived tumor xenografts engrafted in immunodeficient mice have been established and tested for a variety of cancer types [60]. Tumor graft models (also known as patient-derived xenografts or PDXs) are based on the transfer of primary tumors directly from the patient into an immunodeficient mouse. Because PDX mice are derived from human tumors, they offer a tool for developing anticancer therapies and personalized medicine for patients with cancer [63]. Though, they require surgery, not always available in all animal laboratories. In addition, as the exact number of cells required for inoculation is unknown, tumor inoculation is difficult to perform. However, they reflect tumor heterogeneity and do not require mincing and subculturing [63]. Regrettably, no cell line derived from this xenograft is available for *in vitro* experiments [60]. In the meantime, the use of a panel of tumor models of NCI-H295R, SJ-ACC3, and MUC-1 xenograft models could help the clinical translation of therapeutic regimens in the future (Table 1). In fact, in a long-term treatment regimen, this panel displayed model-dependent differences in therapeutic outcomes with specific chemotherapeutic regimens again reflecting the high heterogeneity of ACC patients [75].

### 4.1. YI Mouse Cell Line

The initial model of the ACC originated from LAF1 mice by exposure to the irradiation of a test atomic bomb explosion. Cohen et al. succeeded to transplant the generated ACC into mice, and the transplanted tumor caused the malignant phenotype. By necropsy, they showed numerous small lung metastases characterized by atrophy of the zona glomerulosa and confirmed by the coexisting hypernatremia and polyuria, with early drop in levels of eosinophils. However, no significant change in the mouse pituitary bodies of the tumor-bearing mice was observed, suggesting the model's inability to secrete adrenocortical hormones such as cortisone and hydrocortisone [76]. To address this shortage, a clone secreting adrenocortical hormones and able to cause the malignant phenotype in the LAF1 mouse strain *in vivo* was generated from the later [64, 66]. This offered a huge opportunity at that time when a suitable human model of ACCs was uncommon. In fact, this model has been used by many investigators [65]. However, the YI line poorly mimics human ACC as it presents uncommon properties in the growth, morphology, or adrenocortical hormones involved in steroidogenesis [65].

### 4.2. NCI-H295R

NCI-H295R is a pluripotent ACC cell line established by Gazdar et al. from a carcinoma of the adrenal cortex of a 48-years-old female patient diagnosed with ACC [65, 77]. The cell line is a suitable model of hyperaldosteronism in relation to different culture conditions that retain the ability to produce adrenal androgens [57, 59, 65]. NCI-H295R cells predominantly secrete cortisol, while aldosterone and other steroids are released at much lower concentrations. Indeed, aldosterone output specifically increases in response to different stimuli such as potassium ion, ACTH, and angiotensin II in a dose-dependent manner [59, 65]. Moreover, the cell line has proved value in studying regulation, metabolic pathways, and enzymes involved in steroid formation and secretion [65]. Various sets of cells growing rapidly and with better monolayer attachment have been developed based on the serum supplement used for growth to mitigate the slow growth rates and easy detachment of the original NCI-H295 strains. This includes H295R-S1, H295R-S2, and H295R-S3 [77, 78]. NCI-H295R-derived spheroids release higher aldosterone concentration resulting from higher expression levels of the steroidogenic enzymes steroidogenic acute regulatory protein (StAR), 3*β*-hydroxysteroid dehydrogenase (3*β*-HSD), cytochrome (CYP) 17, SF-1 (steroidogenic factor 1), and the melanocortin 2 (MC2) receptor and the presence of epithelial growth factor (EGF) and fibroblast growth factor (FGF) in the culture medium [59]. As well, the human adrenocortical tumor H295 cell line is a model to evaluate mechanisms controlling CYP19 and CYP21 steroid production [71]. Xenografts produced all three classes of adrenal steroids, with the preferential production of androgens of the Δ4 pathway. The NCI-H295R xenograft model induced after subcutaneous injection of 6 × 10^6^ cells into female nude mice displayed high levels of plasma steroid similar to that of the original patient tumor with a take-on rate in the range of 90% and a medium doubling time of 12 days [70]. The H295R xenograft model is a good model of human ACC, as it mimics dysregulation of the IGF system usually found in these tumors. It also produces IGF-binding protein-2 and steroids that can be used as tumor markers for preclinical evaluation of existing clinical treatment regimens [69, 70, 79–81]. The subcutaneous NCI-H295R xenografts have also been utilized upon genetic modification to study the role of the beta-catenin pathway in the context of adrenocortical tumorigenesis [68]. This model may be useful for evaluating therapeutic agents [65, 70] as well as for the development of novel therapeutic strategies [68, 69]. Indeed, NCI-H295R is the main cell model used in drug discovery against ACC. For example, abiraterone acetate, a potent inhibitor of 17alpha-hydroxylase/17, 20-lyase, a key enzyme of adrenal steroidogenesis that rapidly decreases cortisol, has been used in *in vitro* and in NCI-H295R xenograft models with interesting results [67]. As well, screening inhibitors of TOP2A overexpressed in ACC, which regulates cellular proliferation and invasion in ACC cells, has successfully been used to identify aclarubicin, a good drug candidate for clinical trials in patients with locally advanced and metastatic ACC [82]. However, the use of the cell model for drug discovery and development suggests a poor reproducibility of therapeutic action between different clones of the most commonly used tumor cell model NCI-H295R [60].

### 4.3. SJ-ACC3

An SJ-ACC3 cell model was established from a tumor of an 11-year-old boy harboring a germline *TP53 G245C* mutation. A subcutaneous xenograft with SJ-ACC3 in immunocompromised CB17 scid−/− mice has been generated [61]. The xenograft maintained the histopathologic and molecular features of the original tumor with endocrine functionality, positivity for inhibin-*α*, keratin 8, synaptophysin, a strong nuclear p53 staining, and a Ki67 index of approximately 60%, with no detectable chromogranin, HMB-45, and S-100. Even though it is tissue based and not cell-line based, SJ-ACC3 represents a quite standardizable tumor model confirmed by successful reimplantation after defreezing. Screening the xenograft for drug responsiveness showed that cisplatin had a potent antitumor effect. However, etoposide, doxorubicin, and a panel of other common cancer drugs had little or no antitumor activity, with the exception of topotecan, which was found to significantly inhibit tumor growth [61]. The authors concluded that topotecan could be a potentially effective agent for treating ACC in childhood [61].

### 4.4. SW-13

SW-13 human Caucasian adrenal cortex adenocarcinoma was derived from biopsy tissue of a small cell carcinoma originating in the adrenal cortex of a 55-year-old Caucasian female [72]. This SW-13 cell line was established by Leibovitz et al. in [72] from an undifferentiated small cell carcinoma of lung [72]. SW-13 can be roughly considered an ACC cell line, as it derives from a small cell carcinoma metastasized in the adrenal gland [60].

SW-13 xenografts displayed important angiogenic pathways in ACC that can be used to identify antitumor agents with VEGF receptor tyrosine kinase inhibition properties [62]. Notably, everolimus alone significantly reduced tumor growth in SW13 xenografts, in accordance with the very high percentage of apoptosis seen in *in vitro* experiments. Moreover, sorafenib plus everolimus simultaneous inhibition of several signaling pathways may be a more effective anticancer, suggesting that antiangiogenic drugs may have considerable therapeutic potential for ACC [62]. As well, nanoparticle albumin-bound paclitaxel (nab-paclitaxel) showed better anti-ACC potency in SW-13 tumor-bearing mice than the adrenolytic substance mitotane [83]. Cyclophosphamide administration in SW-13 xenograft mice selectively kills cancer cells without toxicity to cancer stem cells (CSCs) and thereby provides a practical approach for achieving the enrichment of CSCs in ACC [84]. As well, Wolkersdörfer et al. demonstrated the efficacy of suicide gene therapy, a HSV thymidine kinase expressing adenoviral shuttle, is significantly amplified by viral replication and, in combination with ganciclovir, significantly reduces tumor burden and increases survival time in SW-13 xenografts in nude mice injected with 10 × 10^6^ cells [85]. It is noteworthy that SW-13 cells represent a depot to the adrenal of a primitive lung carcinoma and thus have genetic and molecular background that reflects the tissue origin (i.e., no synthesis of adrenal hormones, harbored mutated *TP53*, and wild-type *CTNNB1*).

### 4.5. MUC-1

Hantel et al. established a novel patient-derived tumor model that can improve clinical prediction of therapeutic strategies for ACC patients. The affected patient had an adrenal mass of 22 cm diagnosed as ACC, which has been used to develop a MUC-1 mouse xenograft. This model showed extraordinary engraftment properties and sustained tumor growth over several passages in the murine host with sustained nuclear SF-1 and cytoplasmic 3*β*-HSD immunopositivity, high vascularization, and proliferation [60]. In contrast to the majority of the adrenal patient-derived xenograft, MUC-1 xenografts showed the engraftment of large solid tumors, marked tumor growth, and the maintenance of pathological and endocrine features. Moreover, a primary cell line was established from explanted MUC-1 xenograft pieces, called MUC-1 cells. Differently to NCI-H295R, MUC-1 cells were not responsive to the current systemic gold standard EDP-M treatment [60]. Moreover, genetic and molecular characteristics of MUC-1 cells revealed a distinct marker profile, if compared with NCI-H295R and SW-13 cells, with nuclear p53 and cytoplasmic *β*-catenin staining. Thereby, MUC-1 cells were recently authenticated and certified as a novel ACC cell line of human origin [60].

### 4.6. CU-ACC 1, 2, and 9

CU-ACC1 cell lines derived from a patient with a perinephric metastasis whose primary tumor secreted aldosterone, while CU-ACC2 cell lines derived from a patient with liver metastasis and Lynch syndrome [73]. CU-ACC1 cells carry a mutation in *CTNNB1* and secreted cortisol, but not aldosterone, while CU-ACC2 cells show a *TP53* mutation and loss of *MSH2*, consistent with the patient's known germline mutation causing Lynch syndrome. Short tandem repeat profiling confirmed consistent matches between human samples and models. Both cell lines can be transfected and transduced with similar growth rates and have been used to create a PDX. RNA sequencing and immunohistochemistry confirmed the expression of adrenal cortex markers in the PDXs and human tumors. These new preclinical models of ACC significantly advance the field by allowing investigation of underlying molecular mechanisms of ACC and the ability to test patient-specific therapeutic targets. These new cell lines replicate two of the known genetic models of ACC [73]. A third ACC PDX, CU-ACC9, was established from a mild cortisol producing 15 cm primary ACC tumor involving adrenal, kidney, and distal pancreas with an inferior vena cava (IVC) thrombus in a 55-year-old female patient [74]. CU-ACC9 tumor tissue carries a *T53 p.R248W* mutation, intermediate microsatellite instability, and loss of *MSH2* and *MSH6* staining, all suggestive of Lynch syndrome. It expresses the common adrenal markers inhibin-*α* and SF-1 and high MELK, a kinase identified as a potential therapeutic target in ACC. This model has successfully been used to test the efficacy of OTSSP167 (MELK inhibitor) in the CU-ACC9 PDX model [74].

## 5. Phytochemicals Used in Preclinical Studies

The use of plant natural compounds in cancer is still a matter of debate since human clinical trials testing their usefulness are very scarce and difficult to accomplish, even if some limited results have been reported [86]. Currently, no data are available on the experimentation of phytochemicals in patients affected by ACC or ACT [87]. Nonetheless, different preclinical works have been published, and this section summarizes the available research on ACT and ACC treated with plant natural compounds (Table 2).

### 5.1. Apigenin

**Figure 2:**
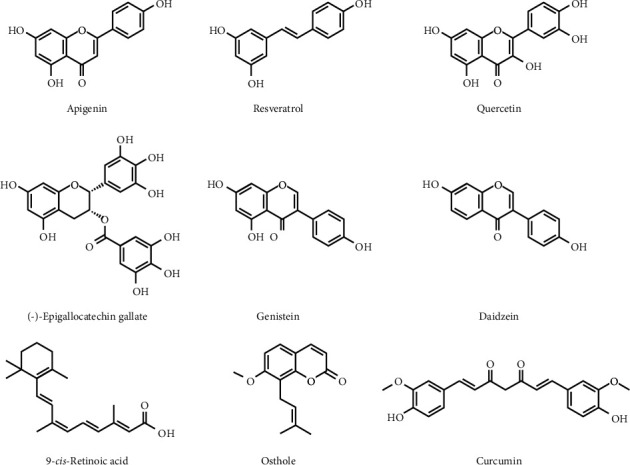
Schematic representation of main phytochemicals used in adrenocortical tumor.

Apigenin (4′,5,7-trihydroxyflavone) (Figure 2) is a naturally occurring flavonoid present in a wide range of plants including *Asteraceae*, *Lamiaceae*, and *Fabaceae* families [105, 106]. Apigenin has been used in traditional medicine for its antioxidant, anti-inflammatory [107], antibacterial, and antiviral activities [108], in addition to its capacity in reducing blood pressure [109]. Furthermore, apigenin was proven to have tumor suppression efficacy via different signaling pathways including NF-*κ*B, PI3K/Akt, JAK/STATs, AMPK, Wnt/*β*-catenin, MAPK/ERK, and JNK and could inhibit cell cycle progression and cell migration and invasion. It could also trigger immune response, autophagy, and cell apoptosis [110]. The protective role of apigenin against multiple types of cancer has been widely reported, for example, in breast [111], cervical [112], colon [113], leukemia [114], lung [115], prostate [116], skin [117], thyroid [118], endometrial [119], neuroblastoma [120], and adrenocortical cancers [94].

Laboratory studies revealed an increased expression of Wnt, frizzle, or lymphoid enhancer factor (LEF)/T cell factor (TCF) in patients with adrenocortical tumors [121]. Apigenin was found to significantly reduce the amount of total, cytoplasmic, and nuclear *β*-catenin and the expression of Wnt target genes in a dose-dependent manner, thereby suppressing cell proliferation, migration, and invasion of cancer cells [122]. Lin et al. also showed that the autolysosomal degradation of *β*-catenin induced by apigenin occurred via inhibition of the Akt/mTOR signaling pathway at concentrations higher than 10 *μ*M [122]. Sanderson *et al.* found that flavonoids was potent aromatase inhibitors and thus increased intracellular cAMP concentrations [122]. Results also indicated that cells exposed to apigenin demonstrated decreased cortisol production and 3*β*-HSD II and P450c21 activity at a concentration of 12.5 *μ*M [88]. More recently, apigenin was shown to downregulate the expression levels of 3*β*-HSD, CYP17, and CYP21 mRNA, an effect demonstrated by increasing levels of pregnenolone and 17*α*-hydroxyprogesterone in forskolin-stimulated NCI-H295R cells [89]. In addition, apigenin and quercetin (30 to 100 *μ*M) induced cytotoxicity in H295R cells, probably causing the decline of *CYP11B1* expression [90]. Thus, this flavonoid could be potentially useful in patients with excessive steroid hormone production, including ACT.

### 5.2. Resveratrol

Resveratrol (trans-3,5,4′-trihydroxysilbene) (Figure 2) is a stilbenoid natural polyphenol found in more than 70 plant species such as tea, pomegranate, berries, and cocoa beans, but it is highly concentrated in the skin and seeds of red grapes [123]. Resveratrol was reported to exhibit many therapeutic benefits including antioxidant and anti-inflammatory properties [124], cardiovascular and neurological protective effects [125, 126], and immunomodulatory properties [127]. Its anticancer properties have been confirmed by several *in vitro* and *in vivo* studies, which showed that resveratrol is able to induce cell cycle arrest, differentiation, and apoptosis and to inhibit cancer cells proliferation [128]. Resveratrol is believed to target the IGF-1R/Akt/Wnt signaling pathway together with downregulation of Bcl-2 expression, activation of caspase-3 and 9, and modulation of p53 expression [129]. Resveratrol can also act as a histone deacetylase inhibitor, decreasing proliferation and inducing apoptosis and autophagy in cancer cells [130]. *In vitro* studies showed that resveratrol could enhance the efficacy of radiotherapy or chemotherapy [131]. Marti *et al.* found that resveratrol (5 *μ*M) reduced protein expression and enzyme activities of CYP17 and CYP21 in H295R cells. Alterations of *CYP17* and *CYP21* genes are associated to rare forms of congenital adrenal hyperplasia, a disease characterized by excessive or deficient production of sex steroids [91]. They also proved that only *SIRT3* mRNA expression was altered by resveratrol associated with the inhibition of PKB/Akt signaling pathway, and thus the authors suggested that resveratrol could reduce androgen production in H295R cells via a sirtuin-dependent mechanism [91]. On the same line, Supornsilchai and collaborators in primary rat adrenocortical cell cultures (*in vitro* and *ex vivo*) showed that the polyphenol (50 *μ*M) inhibited corticosterone production by targeting cytochrome P450 c21-hydroxylase [92]. Moreover, resveratrol (and daidzein) at 10 *μ*M slightly increased cortisol production without affecting *CYP11B1* gene expression or CYP11B1 enzyme activity [90], an enzyme that catalyzes the final conversion of cortisol from 11-deoxycortisol in the biosynthetic pathway. The author suggested that resveratrol (and daidzein) altered cortisol biosynthesis at an earlier step probably acting on CYP17A1, as the time course of substrate-supported cortisol synthesis showed no increase on cortisol production in H295R-treated cells [90]. Similarly, resveratrol and piceatannol (tested for 24 h at 1, 5, 10, 25, 50 *μ*M concentrations) induced a secretion decrease of dehydroepiandrosterone, testosterone, and cortisol and an increase of progesterone. Only resveratrol (not piceatannol) was able to inhibit CYP17A1 activity [93]. The authors detected that resveratrol could inhibit the steroid hormone production by targeting CYP17A1, fundamental in the glucocorticoid biosynthetic pathway. These data suggest that resveratrol can have a role in adrenocortical hormonal production and thus could be potentially useful in the treatment of ACT characterized by dysregulated hormonal synthesis.

### 5.3. Quercetin

Quercetin (3,3′,4′,5,7-pentahydroxyflvanone) (Figure 2) is a polyphenolic bioflavonoid generally classified as a flavonol. It is found in a wide variety of fruits and vegetables such as onions, broccoli, berries, and apples as well as tea and red wine [132]. Quercetin appears to have numerous potential beneficial effects mainly long-lasting anti-inflammatory capacities [133]. It was also reported that quercetin possessed antioxidant actions [134], neurological effects [135], and antiviral activities [136] and was also recognized as an anticancer agent [137]. *In vitro* and *in vivo* studies showed that quercetin had the ability to inhibit the growth of cancer cells due to its antioxidant properties, which prevent DNA damage [138]. These properties have been demonstrated through the increase of glutathione peroxidase (GSH), superoxide dismutase (SOD), and catalase (CAT) and the inhibition of lipid peroxides [139]. Furthermore, quercetin seems to inhibit angiogenesis by the inhibition of protein kinase C [140]. It also prevents cancer development at a physiological concentration of 5 *μ*M by upregulating p53 and increasing the expression of Bax [141]. Its supplementation at doses greater than 30 *μ*M can normalize the expression of insulin-like growth factor receptor 1 (IGF-1), Akt, and androgen receptor (AR) [142]. *In vitro* findings also suggested that quercetin could have a role in reversing drug resistance and increasing the effectiveness of some chemotherapeutics [143]. Sanderson *et al.* established that quercetin at 10 *μ*M induced aromatase activity and increased intracellular cAMP concentrations and levels of the cAMP-dependent pII in H295R human adrenocortical carcinoma cells [94]. Quercetin effectively increased aromatase activity (CYP19), a key enzyme involved in the synthesis of estrogens from androgens, differently from apigenin and resveratrol and other flavonoids that are recognized as aromatase inhibitors [144].

### 5.4. Epigallocatechin Gallate

(-)-Epigallocatechin-3-gallate (EGCG) is the major polyphenol in green tea (*Camellia sinensis* [L.] Kuntze) that has potent antioxidant, anti-inflammatory, and anticarcinogenic properties [145]. It is reported that the galloyl moiety of tea catechins (Figure 2) plays a crucial role in the health benefits of tea catechins including lipid-lowering and antiangiogenic effects [146, 147]. Rady *et al.* reviewed the anticancer effects of EGCG and found that concentrations ranging from 10 to 80 *μ*M induced apoptosis and controlled cell proliferation and angiogenesis [148]. These effects were mediated by the suppression of tumor-associated proteins and the modulation of different signaling pathways such as the regulation of NF-*κ*B, Wnt/*β*-catenin, cyclin D1, p21/WAF1/CIP1, p27/KIP1, and cyclin-dependent kinases [149]. Moreover, EGCG (5–20 *μ*M) suppressed Akt and ERK phosphorylation, blocked MAPK (mitogen-activated protein kinase) activity, and enhanced the activation of FOXO transcription factors [150]. Wu et al. investigated the molecular mechanisms and signal pathways of EGCG on the induction of apoptosis in human adrenal NCI-H295 cancer cells [95]. They showed that EGCG induced growth inhibition in a dose- and time-dependent manner and decreased mitochondrial membrane potential while increasing intracellular free Ca^(2+)^ [95]. In addition, the polyphenol decremented the protein levels of Bcl-2, Bcl-xl, xIAP, cIAP, Hsp70, and Hsp90 and augmented the protein expression of Bad, Bax, Fas/CD95, cytochrome c, Apaf-1, AIF, GADD153, GRP78, and caspase-3, 7, 8, and 9. It seems that EGCG could trigger apoptosis through different mechanisms involving the death-receptor, mitochondrial, and ER stress pathways. Its action on different targets is probably cell-type or tissue-type related; nevertheless, more work is needed to elucidate this hint.

### 5.5. Genistein

Genistein (5,7,4′-trihydroxyisoflavone) (Figure 2) is an isoflavone abundantly found in soy and other legumes [151]. It is a selective estrogen receptor modulator (SERM), mainly through ER*β* binding [152]. Besides the specific SERM effects, genistein has also antioxidant properties and was proved to be a specific inhibitor of tyrosine kinases, affecting many signaling pathways such as protein-tyrosine kinase (PTK), Akt, NF-*κ*B, matrix metalloproteinases (MMPs) and Bax/Bcl-2 [153, 154]. Several studies have reported that genistein at low concentrations (5 *μ*M) modulated various steps of cell cycle, apoptosis, angiogenesis, and metastasis. These anticancer effects are mediated through multiple molecular mechanisms including the enhancement of caspase-9 and caspase-3 activities and the inhibition of NF-*κ*B and Wnt/*β*-catenin signaling pathways [155]. It also increased the proapoptotic protein (Bax) and decreased the antiapoptotic protein Bcl-2 [156]. Interestingly, peroxisome proliferator-activated receptors (PPARs) have also emerged as potential therapeutic targets for modulating tumor growth, and genistein at a concentration of ≥5 *μ*M has been documented to promote apoptosis in tumor cells via targeting PPAR*γ* signaling cascade [157]. Genistein (10 *μ*M) has also been shown to decrease the production of cortisol in ACC cells and inhibit adrenocortical 3*β*-hydroxysteroid dehydrogenase and cytochrome P450-21 hydroxylase in H295R cells [88]. Genistein (10 *μ*M) has been demonstrated to increase aromatase activity in the same cell line and increased aromatase mRNA expression with the concurrent utilization of the CYP19A1 promoters 1.3 and II [94, 158].

### 5.6. Daidzein

Daidzein (7-hydroxy-3-(4-hydroxyphenyl)-4H-chromen-4-one) is a naturally occurring isoflavone found mostly in soybeans and other legumes (Figure 2). The inhibitory effects of daidzein (and genistein) on the secretion of cortisol and corticosterone in porcine adrenocortical cells were related to their ability to suppress the activity of 3*β*-hydroxysteroid dehydrogenase (3*β*-HSD), a key enzyme in the synthesis of glucocorticoids and androstenedione [96]. Mesiano et al. showed significant inhibition of both 3*β*-HSD2 and CYP21A2 at 12.5 *μ*M and that of 3*β*-HSD2 at lower concentrations of 1 *μ*M genistein and 3.1 *μ*M daidzein [97]. *In vitro* assays with 1 and 10 *μ*M daidzein (and genistein) showed that the isoflavone significantly inhibited the lyase activity. It was reported that while −70% of 17-hydroxypregnenolone was converted to dehydroepiandrosterone (DHEA) after 24 h in the absence of isoflavones, only −33% was converted to DHEA in the presence of daidzein and −55% in the presence of genistein. Lower concentrations (1 *μ*M) of both isoflavones also resulted in significant inhibition of the lyase activity and in a decrease of androgen biosynthesis [97].

### 5.7. Retinoic Acid

Retinoids control critical physiological events including cell growth, differentiation, reproduction, metabolism, hematopoiesis, and apoptosis [159]. Retinoic acid signaling through retinoid *X* receptor is recognized as a pathogenic pathway in ACC, and ACC has been associated with reduced retinoic acid production [160]. 9-cis retinoic acid (Figure 2) is an active metabolite of vitamin A, which in turn derives from dark-green leafy vegetables and fruits as provitamin A carotenoids, mainly *β*-carotene [161]. In the H295R cell line, 9-cis retinoic acid reduced DNA synthesis in a dose-dependent manner (1 to 20 *μ*M) [98]. The same authors studied the 9-cis retinoic acid's antitumoral effects in ACC [99], and in an *in vitro* experiment, 9-cis retinoic acid inhibited cell proliferation and hormone secretion of H295R cells, while in an *in vivo* experiment, athymic nude mice xenografted with H295R cells corroborated its antitumor activity (reduced tumor weight).

### 5.8. Osthole

Coumarins from the fruit of *Cnidium monnieri* (L.) Cusson were tested in a rat model of “Kidney *Yang* deficiency,” a typical condition in Chinese medicine that shares clinical signs of the glucocorticoid withdrawal syndrome [162]. The authors showed that osthole (Figure 2), a derivative of coumarin found in *Cnidium monnieri*, decreased plasma corticosterone and adrenocorticotropic hormone without affecting the levels of renin, angiotensin II, and aldosterone [163]. Osthole has been also studied in Y1 mouse ACT cells [100, 101]. At 100 and 200 *μ*M, osthole inhibited Y1 cell proliferation and the gene expression of Star, Cyp11a1, Cyp21a1, Hsd3b2, Cyp11b1, Cyp17a1, and *Hsd17b3* were significantly enhanced [101]. Moreover, at 50 *μ*M, the corticosterone level was increased. The same authors observed that in Y1 cells, osthole stimulated corticosterone secretion in a dose- and time-dependent manner [100]. The authors suggested that osthole could regulate adrenal cortex function through increasing the expression of steroidogenic enzymes genes and thus the molecule could be potentially used in higher models to verify its efficacy in ACT.

### 5.9. Curcumin

Curcumin is the main natural polyphenol found in *Curcuma longa* L. that is widely known for its antioxidant, anti-inflammatory, antimutagenic, antimicrobial, and anticancer properties [164]. A derivative of curcumin is EF24 that shows enhanced solubility if compared with the natural polyphenols. EF24 has been studied in SW13 and H295R cell models in association to mitotane, the reference drug for ACC [102]. The authors showed that EF24 had an antiproliferative effect with IC50 of 6.5 *μ*M and 4.9 *μ*M for SW13 and H295R cells, respectively, induced subG0/G1 cell cycle phase, reduced cell migration and colony number, and increased reactive oxygen species. Different signaling pathways were altered after EF24 treatment, such as Wnt/*β*-catenin, NF-*κ*B, MAPK, and PI3k/Akt pathways.

### 5.10. Other Phytochemicals

Eriodictyol is a tetrahydroxyflavanone isolated from *Eriodictyon californicum* (Hook. & Arn.) Torr., while hesperetin is the 4′-methoxy derivative of eriodictyol, naturally occurring as a flavanone glycoside in lemons and sweet oranges [165]. Naringenin is a trihydroxyflavanone widely distributed in several *Citrus* fruits, bergamot, tomatoes, and other fruits [166]. Hasegawa *et al.* proved that eriodictyol, hesperetin, and naringenin significantly reduced deoxycorticosterone and androstenedione levels by the inhibition of 3*β*-HSD [89]. It is suggested that such inhibition could be potentially useful in the treatment of adrenocortical hypersecretive diseases, including adrenocortical tumors.

### 5.11. Whole Plant Extracts

Few works explored the use of crude plant extracts in SW13 and H295R cell models of ACT. One work evaluated a methanolic extract of *Origanum vulgare* L. collected in the wild and found a cytotoxic activity (IC50) value of 0.4 *μ*g/*μ*L for SW13 cells and 0.8 *μ*g/*μ*L for H295R cells [103]. Moreover, colony formation, cell cycle, and morphological changes were performed, together with a prevalence of necrotic process over apoptosis. MAPK and PI3K/Akt pathways were involved in the antiproliferative effect of oregano. Similarly, a second work explored a collected wild mountain *Mentha longifolia* (L.) Hudson extract in the same cell lines [104]. The research showed that the extract exerted antiproliferative effect by MTT assay only in SW13 cells at 0.5 *μ*g/*μ*L, together with an increase in subG0/G1 phase. No reinforced effect was observed when mint was combined with mitotane; nonetheless, the plant extract modulated the MAPK signaling pathway in SW13 cell lines.

The use of plant crude extract could be a promising strategy to fight cancer, as it uses phytocomplexes rather than a single compound or active molecule [167–169]. This line of research can reserve interesting developments because synergy and/or potentiating effects are at the basis of the phytocomplex; thus, phytochemicals can possess greater effects than the sum of the effects of the individual components [170].

## 6. Conclusions and Future Perspectives

Plant natural products are a precious source of complex and innovative compounds, useful in numerous clinical conditions, including cancer. Phytochemicals are also a source of inspiration for new medicines and supplements, given their high biochemical structure variability. Among this broad category, polyphenols are probably the most studied and represented, for example, curcumin, resveratrol, and quercetin; and they reserve remarkable volumes of literature clinical evidence with anticancer activity, at least in the most common forms of human tumors, i.e., lung, mammary, colorectal, and prostate [171]. These findings are a good omen for the success of antineoplastic therapy also in rare and uncommon cancer, such as ACC. Regrettably, available data in literature and presented in this review cannot permit to conclude that plant natural compounds are a real tool to fight ACC. Data are partial and represented only by preclinical experiments. Rather than to be a limit, this should be considered a stimulus to encourage researchers to spend new resources and energy in the field of plant-derived drugs discovery, with a special emphasis on those biochemical compounds with anticancer activity.

## Figures and Tables

**Table 1:** Cell line models of ACT.

Cell line	Features	Similarity to human adrenocortical cancer	Reference
YI	Derived from LAF1 mice, isolated after different *in vivo* passages and then in cell cultures, could secrete some adrenocortical hormones	Poor (alteration in growth, morphology, or adrenocortical hormones)	[64–66]
NCI-H295R (H295, H295R, H295R-S1, H295R-S2, and H295R-S3)	Derived from a carcinoma of the adrenal cortex of a 48-year-old female patient diagnosed with ACC, can produce and secrete human adrenocortical hormones	High (most commonly used cell line), mouse xenograft used	[65, 67–71]
SJ-ACC3	Derived from a tumor of an 11-year-old boy harboring a germline *TP53* G245C mutation	Medium (it resembles genetic childhood ACC), mouse xenograft used	[61]
SW-13	Derived from a metastatic depot in the adrenal cortex of a 55-year-old Caucasian female with a small cell carcinoma (lung), no hormonal secretion	Poor (roughly considered an ACC cell line; however, frequently used as a control cell line), mouse xenograft used	[72]
MUC-1	Derived from an ACC patient with an adrenal mass of 22 cm engrafted in mice and then explanted, genetic and molecular characteristics of MUC-1 cells have a distinct marker profile, if compared with NCI-H295R	High (even if mouse-conditioned), mouse xenograft used	[60]
CU-ACC1	Derived from a patient with a perinephric metastasis whose primary tumor secreted aldosterone	Medium (it resembles aldosterone-producing adenoma)	[73]
CU-ACC2	Derived from a patient with liver metastasis and Lynch syndrome	Low (roughly considered an ACC cell line)	[73]
CU-ACC9	Derived from a cortisol-producing ACC involving adrenal, kidney, and distal pancreas with an inferior vena cava (IVC) thrombus in a 55-year-old female	Medium (it resembles cortisol-producing ACC, suggestive of Lynch syndrome)	[74]

**Table 2:** Phytochemicals used in preclinical studies.

Phytochemical	Experimental model	Doses	Proposed mechanism of action	Reference
Apigenin	H295R cells	12.5 *μ*M	Decreased cortisol production and 3*β*-HSD II and P450c21 activity	[88]
	NCI-H295R cells (forskolin-stimulated)	30 *μ*M	Increased levels of pregnenolone and 17*α*-hydroxyprogesterone and downregulated the expression levels of 3*β*-HSD, CYP17, and CYP21 mRNA, reduced deoxycorticosterone and androstenedione levels, suggesting inhibition of 3*β*-HSD	[89]
	H295R cells	30 *μ*M	Decreased *CYP11B1* expression, increased cytotoxicity	[90]

Resveratrol	H295R cells	5 *μ*M	Reduced protein expression and enzyme activities of CYP17 and CYP21, inhibited PKB/Akt signaling pathway through sirtuin (*SIRT3* mRNA modification)	[91]
	Primary rat adrenocortical cell cultures (*in vitro* and *ex vivo*)	50 *μ*M	Inhibited corticosterone production by targeting cytochrome P450 c21-hydroxylase	[92]
	H295R cells	10 *μ*M	Increased cortisol production without affecting *CYP11B1* gene expression or CYP11B1 enzyme activity	[90]
	H295R cells	1, 5, 10, 25, 50 *μ*M	Induced a secretion decrease of dehydroepiandrosterone, testosterone, and cortisol, and an increase of progesterone	[93]

Quercetin	H295R cells	10 *μ*M	Induced aromatase activity and increased intracellular cAMP concentrations and levels of the cAMP-dependent pII	[94]

Epigallocatechin gallate	NCI-H295 cells	20 *μ*M	Induced growth inhibition in a dose- and time-dependent manner, decreased mitochondrial membrane potential, increased intracellular free Ca^(2+)^, decreased the protein levels of Bcl-2, Bcl-xl, xIAP, cIAP, Hsp70, and Hsp90, increased the protein expression of Bad, Bax, Fas/CD95, cytochrome c, Apaf-1, AIF, GADD153, GRP78, and caspase-3, 7, 8, and 9	[95]

Genistein	H295R cells	10 *μ*M	Decreased cortisol production and inhibited adrenocortical 3*β*-hydroxysteroid dehydrogenase and cytochrome P450-21 hydroxylase	[88]
	H295R cells	10 *μ*M	Induced aromatase activity and increased intracellular cAMP concentrations and levels of the cAMP-dependent pII	[94]
	Porcine adrenocortical cells	10 *μ*M	Inhibited secretion of cortisol and corticosterone via progesterone synthesis inhibition (through the suppression 3*β*-hydroxysteroid dehydrogenase (3*β*-HSD) activity)	[96]
	H295 cells	1 *μ*M	Inhibited 3*β*-HSD2, CYP21A2, and 3*β*-HSD2, inhibited *in vitro* the lyase activity, reduced 17-hydroxypregnenolone conversion to dehydroepiandrosterone (DHEA), decreased biosynthesis of androgens	[97]

Daidzein	Porcine adrenocortical cells	10 *μ*M	Inhibited secretion of cortisol and corticosterone via progesterone synthesis inhibition (through the suppression 3*β*-hydroxysteroid dehydrogenase (3*β*-HSD) activity)	[96]
	H295 cells	3.1 *μ*M	Inhibited 3*β*-HSD2, CYP21A2, and 3*β*-HSD2, inhibited *in vitro* the lyase activity, reduced 17-hydroxypregnenolone conversion to dehydroepiandrosterone (DHEA), decreased biosynthesis of androgens	[97]

Retinoic acid	H295R cells	1–20 *μ*M	Reduced DNA synthesis in a dose-dependent manner	[98]
	NCI-H295R cells and athymic nude mice xenografted with H295R cells	10, 25, 50, 75, 100 *μ*M	Inhibited cell proliferation and hormone secretion in cell models, reduced tumor weight in animal models, implication of cell cycle regulation by network analysis	[99]

Osthole	Y1 mouse cells	100–200 *μ*M	Inhibited cell proliferation, enhanced gene expression of Star, Cyp11a1, Cyp21a1, Hsd3b2, Cyp11b1, Cyp17a1, and *Hsd17b3*, increased corticosterone secretion in a dose- and time-dependent manner	[100, 101]

Curcumin (curcumin derivative EF24)	SW13 and H295R cells	IC50 of 6.5 and 4.9 *μ*M, respectively	Induced subG0/G1 cell cycle phase, reduced cell migration and colony number, increased reactive oxygen species, involvement of Wnt/*β*-catenin, NF-*κ*B, MAPK, and PI3k/Akt pathways	[102]

Other compounds: eriodictyol, hesperetin, and naringenin	NCI-H295R cells (forskolin-stimulated)	30 *μ*M	All reduced deoxycorticosterone and androstenedione levels by the inhibition of 3*β*-HSD	[89]

Whole plant extracts	SW13 and H295R cells	IC50 of 0.4 *μ*g/*μ*L and 0.8 *μ*g/*μ*L, respectively	Methanolic extract of *Origanum vulgare* impacted on colony formation, cell cycle, and morphological changes, with a prevalence of necrotic process over apoptosis, MAPK, and PI3K/Akt pathways involvement	[103]
	SW13 and H295R cells	0.5 *μ*g/*μ*L in SW13	Methanolic extract of *Mentha longifolia* increased subG0/G1 phase, induced cytotoxicity, in both cell lines. In SW13 cells, the mixture modulated the MAPK pathway	[104]

## Data Availability

Data are available on request to the corresponding author.
